# Biomechanical analysis of a centralization procedure for extruded lateral meniscus after meniscectomy in porcine knee joints

**DOI:** 10.1002/jor.25146

**Published:** 2021-08-05

**Authors:** Yuji Kohno, Hideyuki Koga, Nobutake Ozeki, Junpei Matsuda, Mitsuru Mizuno, Hisako Katano, Ichiro Sekiya

**Affiliations:** ^1^ Center for Stem Cells and Regenerative Medicine Tokyo Medical and Dental University Tokyo Japan; ^2^ Department of Joint Surgery and Sports Medicine Tokyo Medical and Dental University Tokyo Japan

**Keywords:** centralization with capsular advancement, load distribution analysis, meniscal extrusion after meniscectomy

## Abstract

The recently developed arthroscopic centralization for lateral meniscal extrusion has obtained satisfactory short‐term clinical and radiological results and improves the meniscus biomechanical properties. However, the effectiveness of treatment for meniscus extrusion after partial meniscectomy still requires elucidation. This study investigated the effect of centralization with modifications from a mechanical viewpoint. Porcine knee joints (*N* = 6) were set in a universal tester under the following conditions: (1) Intact; (2) Meniscectomy: Inner half of the posterior half meniscus was removed; (3) Extrusion: Posterior meniscus was dislocated laterally by transecting the posterior root and the meniscotibial ligament; (4) Centralization‐1: Centralization procedure using one anchor; (5) Centralization‐2: Centralization procedure using two anchors; and (6) Centralization‐ad: Centralization with capsular advancement using two anchors. Load distributions and contact pressure in the meniscus and tibial cartilage were evaluated with an axial compressive force of 200 N. After meniscectomy, the tibial cartilage load increased and that of the medial margin of the posterior part of the meniscus decreased. When the meniscus was extruded, the load was concentrated only on the tibial cartilage. Centralization‐1 increased the load on the meniscus, while Centralization‐2 further increased the meniscus load but decreased the tibial cartilage load. Centralization‐ad further decreased the load on the tibial plateau. The average contact pressure of the tibial cartilage was significantly higher in the Extrusion group than in the Intact group or the Centralization‐ad group. From a biomechanical viewpoint, centralization with capsular advancement was the most effective of the tested procedures for treatment for an extruded meniscus after partial meniscectomy.

**Abbreviations**
CI: confidence interval
LCL: lateral collateral ligament
LM: lateral meniscus
OA: osteoarthritis

## 1 INTRODUCTION

Meniscal extrusion induces dysfunction of load distribution^1^, ^2^, ^3^ and is often observed after meniscectomy^4^, ^5^ and meniscus root tears,^6^ as well as with aging.^7^, ^8^, ^9^ Even if the meniscectomy is not extended to the popliteal hiatus, the fragility of the attachment site of the meniscotibial capsular ligament just anterior to the popliteal hiatus results in loosening of the capsular ligament and leads to extrusion.^10^ This extrusion correlates with symptoms of the knee joints, especially knee pain, and initiates knee osteoarthritis (OA) as the extrusion progresses.^11^, ^12^, ^13^ An association has been reported between a poorer clinical outcome after partial meniscectomy and a greater severity of meniscal extrusion.^5^ Therefore, restoring the loss of function of the meniscus can improve the symptoms and delay knee OA progression.^14^

An arthroscopic centralization technique has been developed to centralize the mid‐body of the lateral meniscus (LM) to reduce/prevent extrusion.^15^ The advantage of this technique is that the function of the LM in distributing loads can be restored if the mid‐body of the meniscus is retained. Therefore, this technique is applicable to cases with osteochondral injury or OA progression caused by extrusion of the LM after partial meniscectomy, as well as cases with symptomatic lateral discoid meniscus where meniscoplasty is planned. This procedure has shown satisfactory short‐term clinical and radiological results^16^, ^17^ and improves the biomechanical properties of the meniscus for load distribution,^18^, ^19^, ^20^, ^21^ making it one of the potential surgical options that can prevent the progression of OA due to meniscal extrusion. However, treatment of meniscus extrusion remains difficult after partial meniscectomy, the major surgical treatment for the meniscus,^22^ despite recent improvements in the techniques for meniscus repair.^23^

Meniscoplasty of the LM by capsular advancement has recently been reported as a treatment for meniscus extrusion with meniscus defects. With this technique, the meniscotibial capsule is released from the tibia and advanced with the remaining meniscus onto the rim of the tibial plateau to reform a meniscus‐like configuration. This surgical intervention was shown to improve clinical and radiographic outcomes at a 2‐year follow‐up in patients with lateral compartment OA attributed to LM defects.^24^ The purpose of the present study was to investigate the effect of centralization with modifications from the mechanical viewpoint using an existing porcine knee joint model.^18^, ^21^

## 2 METHODS

### 2.1 Porcine knee joints

We used the knee joints of approximately 100 kg of 6‐month‐old commercial pork pigs (strain and gender unknown; Tokyo Shibaura Zouki). The porcine knee joints were fresh‐frozen and only right‐side joints. Any knees with a damaged meniscus or cartilage were excluded. The lateral compartments were analyzed in six right knees.

### 2.2 Experimental setup

The experimental setup was described previously.^18^, ^21^ The muscles around the knee joint were removed before cutting the bone. In brief, the femur bone was cut obliquely at 45° at 7 cm proximal from the joint and the tibia bone was cut horizontally at 3 cm distal to the joint. The resulting joint was then fixed to a tester using polymethylmethacrylate. The meniscotibial capsule and the medial collateral ligament were preserved, whereas the lateral collateral ligament (LCL) and surrounding joint capsule were resected to insert the sensor seat. The anterior cruciate ligament, posterior cruciate ligament, medial meniscus, and LM were preserved in the joint.

**Figure 1:**
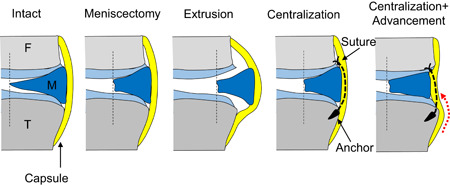
Experimental settings. Intact—intact lateral meniscus (F: femur, M: meniscus, T: tibia); Meniscectomy—inner half of the posterior half meniscus was removed; Extrusion—posterior meniscus was dislocated laterally by transecting the posterior root and the meniscotibial ligament; Centralization—centralization procedure using one or two anchors; Centralization + advancement—centralization with capsular advancement (red dotted line) using two anchors, which moved the inner margin of the meniscus to the original position of the intact meniscus [Color figure can be viewed at wileyonlinelibrary.com]

**Figure 2:**
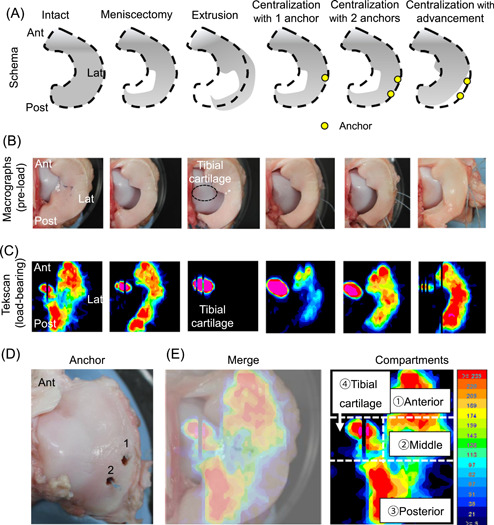
Load distribution analyzed with a pressure mapping sensor system. (A) Schema of the experiments from an overhead view. (B) Pre‐load macrographs of the meniscus from the overhead view. (C) Representative load distribution at an axial compressive force of 200 N. (D) Macrograph of first and second anchor placements on the tibia. (E) Tibial cartilage with lateral meniscus; superposed image of the load distribution and a macro picture, with the lateral tibial surface divided into four compartments [Color figure can be viewed at wileyonlinelibrary.com]

The mechanical setup was as follows: (1) Intact; (2) Meniscectomy—the inner half of the posterior portion of the meniscus was removed; (3) Extrusion—after meniscectomy, the posterior portion of the meniscus was displaced laterally by transecting a 1‐cm width of the posterior root of the LM and the meniscotibial ligament; (4) Centralization with one anchor (Centralization‐1)—a 1.4‐mm soft anchor (JuggerKnot, Zimmer Biomet,) was inserted into the lateral tibial plateau at 2 cm anterior to the popliteal hiatus, and sutures were passed through the capsule and tied to the centralized meniscus; (5) Centralization with two anchors (Centralization‐2)—after centralization with one anchor, another 1.4 mm soft anchor was inserted into the lateral tibial plateau at 1 cm posterior to the first anchor, and the same procedure was repeated for centralization; and (6) Centralization with capsular advancement (Centralization‐ad)—the two sutures used for centralization were unknotted and the capsule attached to the meniscus was released from the tibia to mobilize the capsule to the inner side of the joint; sutures and tying were completed so that the inner margin of the meniscectomized meniscus was advanced to the original position of the intact meniscus (Figures 1 and 2A). Pre‐load macrographs of the meniscus and their schema are shown in Figure 2B. An axial compressive force of 200 N was applied in each setting.^18^, ^21^, ^25^

### 2.3 Contact area and force measurements

The load‐distributing force on the lateral compartment was quantified using a pressure mapping sensor system that enabled the measurement of the real‐time force and contact area (Tekscan, Inc.).^26^, ^27^, ^28^ The sensor was placed on the femoral side of the LM and recorded the load distribution, as well as average contact pressure, maximum load, and contact area. The data were analyzed with MATLAB® (MathWorks).

### 2.4 Statistics

The Friedman one‐way non‐parametric test and Dunn's multiple comparisons test were used as post hoc tests and calculated using Prism 8 software (GraphPad Inc.). *P* values less than 0.05 were considered statistically significant. All data were shown as means with 95% confidence intervals (CIs).

## 3 RESULTS

Application of a 200‐N loading force to the joint resulted in a load distribution on the meniscus and tibial cartilage in the intact knee (Figure 2C). Meniscectomy of the inner half of the posterior portion of the meniscus increased the load of the tibial cartilage increased and decreased the load of the medial margin of posterior part of the meniscus. In addition, meniscus extrusion caused a load concentration only on the tibial cartilage. Centralization with one anchor restored the load pressure on the meniscus, and this was further improved with two anchors with further reduction of the load distribution on the tibial cartilage. Centralization with capsular advancement further decreased the load on the tibial plateau.

**Figure 3:**
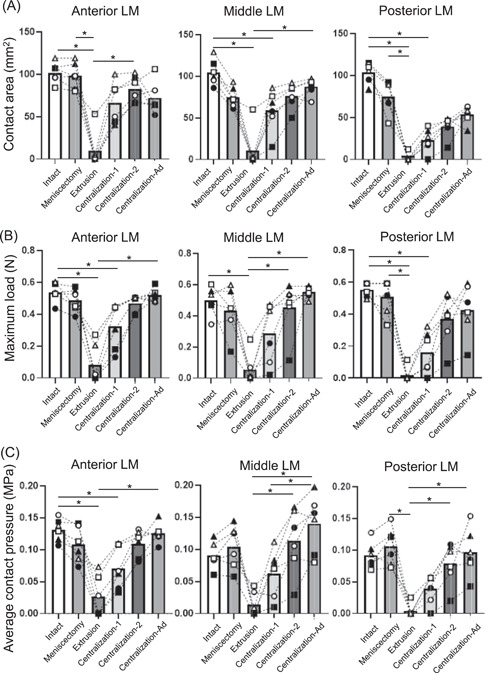
Quantitative analyses in the anterior, middle, and posterior lateral meniscus (LM). (A) Contact area. (B) Maximum load. The value measured in one cell (0.3 × 0.3 mm^2^) of the sensor. (C) Average contact pressure. The average values with 95% CI are shown (*n* = 6). *p < 0.05

Quantitative evaluations were conducted by dividing the lateral compartment into the anterior LM, the middle LM, the posterior LM, and the tibial cartilage areas (Figure 2D). The contact area significantly decreased after extrusion in the anterior, middle, and posterior LM. By contrast, this area significantly increased after centralization with advancement in the middle LM, whereas none of the centralizations fully restored the contact area in the anterior and posterior LM (Figure 3A and Table S1).

The maximum load decreased after meniscectomy and further decreased significantly after extrusion, whereas it increased after centralization step by step in every part of the LM. However, the maximum load was not fully restored even after centralization with advancement in the posterior LM (Figure 3B and Table S2).

**Figure 4:**
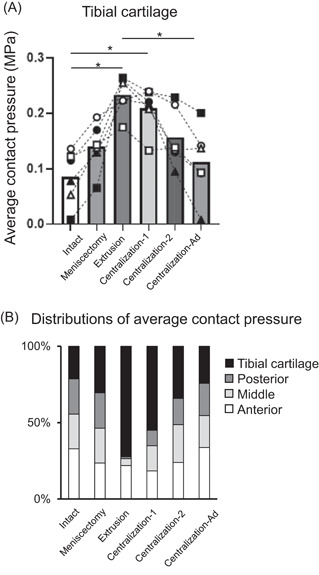
Quantitative analyses of average contact pressure on the lateral tibial cartilage. (A) Average contact pressure on the lateral tibial cartilage. The average values with 95% CI are shown (*n* = 6). *p < 0.05. (B) Distributions of average contact pressure on the lateral compartment

Similar results to those for maximum load were obtained for the average contact pressure in every part of LM. This pressure was fully restored after centralization with advancement, even in the posterior LM (Figure 3C and Table S3). In the tibial cartilage, the average contact pressure significantly increased after extrusion, whereas it significantly decreased after centralization with advancement (Figure 4A and Table S4). The distribution of the average contact pressure showed a similar pattern between the intact group and the group after centralization with advancement (Figure 4B).

## 4 DISCUSSION

This biomechanical study showed that the centralization procedure for meniscus extrusion after partial meniscectomy can reduce extrusion of the LM; however, this procedure was not sufficient for restoration of the load distribution function of the LM. Conversely, the centralization with advancement significantly decreased the average contact pressure on the tibial cartilage and restored a distribution pattern similar to that of the intact knee joint.

Meniscal defects after meniscectomy of the LM are one of the main causes of secondary knee OA of the lateral compartment. Koga et al.^24^ reported that meniscoplasty of the LM by capsular advancement improved the clinical and radiographic outcomes at the 2‐year follow‐up in patients with lateral compartment OA attributed to lateral meniscal defects. However, the biomechanics of this procedure remains unclear. Therefore, we created our extruded LM model after partial meniscectomy and analyzed the centralization procedure using one or two anchors, or two anchors with capsular advancement in porcine knee joints.

Centralization with one or two anchors did not fully recover the contact area in the LM; however, centralization with advancement restored both the maximum load and the contact pressure. In the middle LM, the contact area, maximum load, and contact pressure increased after centralization with one anchor and these were further increased with two anchors, whereas they were fully restored after centralization with advancement. In the posterior LM, none of the centralization groups showed full recovery of the contact area and maximum load, as only the contact pressure was recovered after centralization with advancement. These findings indicated that centralization with one or two anchors was useful for restoration of the meniscus function to some extent; however, adding the procedure of capsular advancement was more effective for the recovery of most of the meniscus functions in the knee with meniscus extrusion after partial meniscectomy.

In regard to the tibial cartilage, meniscus extrusion significantly increased the average contact pressure, whereas centralization with advancement significantly decreased it and provided a redistribution of the average contact pressure similar to that in the intact knee. This finding indicates that this procedure would prevent the progression of cartilage degeneration due to the dysfunction of the meniscus.

The contact area and maximum load decreased in extrusion were not fully restored in the posterior LM, even after centralization with advancement. This could possibly reflect the fact that a 1‐cm width of the posterior root deficiency was left untreated. These results suggest that a hoop function should also be reconstructed, if possible, for full restoration of the load distribution function of the posterior LM. Even so, this procedure fully recovered the contact pressure in the posterior LM and decreased the contact pressure in the tibial cartilage, confirming the effectiveness of the procedure that combined capsular advancement with centralization. Clinically, if the meniscal defect is large, then meniscal reconstruction using an autologous tendon graft^29^, ^30^ or transplantation using an allogeneic meniscus^31^ are usually chosen for treatment options. The centralization technique represents another possible option that could accompany these techniques. Daney et al.^19^ reported the use of a transtibial centralization technique to minimize meniscus extrusion. Future anticipated treatment options would also include transplantation of mesenchymal stem cells in addition to meniscus repair^32^ or transplantation of heterogeneous menisci using decellularization technology.^33^

Several studies with comparable findings to those of the current study have recently been reported. For example, Nakamura et al.^20^ used the centralization procedure in an ACL‐reconstructed porcine knee with an irreparable LM defect to evaluate the effects on knee biomechanics. Daney et al.^19^ also measured meniscal extrusion and tibiofemoral contact mechanics at the medial compartment in human cadaveric knees. Both of these studies confirmed the effectiveness of centralization, in agreement with the current study. Several biomechanical studies have also investigated the load distribution of a torn meniscus using a pig model. Tachibana et al.^25^ reported that a radial tear of 100% width involving the rim significantly decreased the in situ force of the LM and caused a medial shift and valgus rotation of the tibia. Similarly, Ohori et al.^34^ reported a detrimental effect of a complete LM radial tear on the load distribution and transmission functions, with the greatest effect observed for the posterior root tear, followed by the posterior portion tear, and then the middle portion tear in the deep‐flexed position. They claimed that complete radial tears of the meniscus, especially at the posterior root, should be repaired to restore biomechanical function. As previously mentioned, a hoop function should be reconstructed for full restoration of the load distribution function of the posterior LM in cases of meniscal tear or extrusion.

The knee flexion angle was set at 45° and 200 N of an axial compressive force was applied at each setting, based on previous studies^18^, ^21^ that analyzed the biomechanical effects of centralization in a porcine meniscus extrusion model by resection of the posterior root of the LM and posterior capsule. The other experimental settings were also the same as in these studies. The difference between the previous studies and the present study was that the previous study analyzed the biomechanics at 30°, 60°, and 90°, as well as at 45°, which is the physiological extension angle of pigs.^35^ The centralization procedure could reduce extrusion of the LM and restore the load distribution function at all angles, and no significant differences were detected in the contact area and contact pressure at different angles. For this reason, only a knee flexion angle of 45° was tested in the current study. The choice of an axial compressive force of 200 N was made because previous reports indicated that this force would be large enough to yield clinically significant findings.^18^, ^21^

The Tekscan device is one of the most popular biomechanical testing devices and is able to measure the real‐time contact forces of the knee joint.^26^, ^27^, ^28^ We used the Tekscan Sensor Model 5027, and the pressure sensor film was calibrated and equilibrated using the Tekscan pressure calibration unit and correction software for each specimen. The maximum load (N) was the value measured in one cell (0.3 × 0.3 mm^2^) of the sensor. The curved interface might possibly impact the measurements because the sensor was placed between the femoral cartilage and the LM. However, the sensor did not move throughout the testing because it was stabilized by the pressure within the joint space. We also repeatedly added saline mist throughout the experiment to avoid wrinkling of the film with time due to the dry environment.^18^ Nevertheless, the cells in the sensor film did not react to the loading force. Therefore, the data set was adjusted with respect to the neighboring cells.

This study had some limitations. One was that the LCL was cut to insert a sensor from the lateral side, thereby raising concerns that instability caused by LCL deficiency could have affected the results. Another was that all the experiments involving cutting used the same sequences in this study; however, to evaluate the effect of meniscectomy, extrusion, and centralization, other sequences of cutting should be tested. A pressure mapping sensor was also inserted between the femoral cartilage and the LM, rather than between the LM and the tibial cartilage, as the latter placement would have impaired the load‐distribution measurements for the entire tibial cartilage. However, the knee joint was stabilized, and the loading force was applied in the vertical direction, so the comparison of the evaluated values under intact, meniscectomy, extrusion, and centralization settings therefore provide important information. This was a study conducted at time 0, and a biomechanical study to show the effect of centralization after cyclic loading is a future task.

## 5 CONCLUSIONS

The centralization procedure could reduce extrusion of the LM and restore the load distribution function of the LM in a porcine model. From a biomechanical viewpoint, centralization with capsular advancement was the most effective among the tested centralization procedures as a treatment for an extruded meniscus after partial meniscectomy.

## AUTHOR CONTRIBUTIONS

Yuji Kohno: Data collection and draft writing. Hideyuki Koga: Conception, manuscript editing, and critical advice. Nobutake Ozeki: Study design, data analysis, and critical advice, and final approval of the article. Junpei Matsuda: Mechanical setting and interpretation of data. Mitsuru Mizuno: Data collection. Hisako Katano: Data interpretation. Ichiro Sekiya: Interpretation of data, major manuscript writing, and revised manuscript writing.

## Supporting information

Supporting information.

Supporting information.

Supporting information.

Supporting information.
