# Supplementary material for: Biomechanical analysis of a centralization procedure for extruded lateral meniscus after meniscectomy in porcine knee joints
Source: J Orthop Res. 2021 Aug 5;40(5):1097–103. doi: 10.1002/jor.25146 (PMC9292650; doi:10.1002/jor.25146)
Supplement: Supplementary file 1 — Supporting information. [file JOR-40-1097-s004.docx]

**Supplementary Table 1. Contact area for the anterior, middle, and posterior lateral meniscus (LM).**

|  | **Contact area (mm^2^)** | | |
| --- | --- | --- | --- |
|  | **Anterior** | **Middle** | **Posterior** |
| **Intact** | 101.7  (91.9~111.4) | 104.8  (92.6~117.1) | 103.7  (93.8~113.6) |
| **Meniscectomy** | 98.0  (85.5~110.5) | 74.7  (65.0~84.4) | 75.0  (59.8~90.2) |
| **Extrusion** | 9.8^abc^  (-7.2~26.8) | 10.7^ad^  (-8.7~30.0) | 2.0^ab^  (-1.9~5.9) |
| **Centralization with 1 anchor** | 66.2  (46.4~85.9) | 59.2^e^  (39.3~79.0) | 22.8^e^  (11.5~34.2) |
| **Centralization with 2 anchors** | 82.8  (70.7~95.0) | 76.3  (64.2~88.5) | 39.2  (28.9~49.4) |
| **Centralization with advancement** | 72.0  (56.8~87.2) | 87.7  (79.4~95.9) | 53.3  (44.8~61.9) |

Average values with 95% CI for 6 samples are shown.

^a^ p < 0.05 between the Intact group and the Extrusion group

^b^ p < 0.05 between the Meniscectomy group and the Extrusion group

^c^ p < 0.05 between the Centralization-2 group and the Extrusion group

^d^ p < 0.05 between the Centralization-ad group and the Extrusion group

^e^ p < 0.05 between the Intact group and the Centralization-1 group
