# Supplementary material for: Biomechanical analysis of a centralization procedure for extruded lateral meniscus after meniscectomy in porcine knee joints
Source: J Orthop Res. 2021 Aug 5;40(5):1097–103. doi: 10.1002/jor.25146 (PMC9292650; doi:10.1002/jor.25146)
Supplement: Supplementary file 2 — Supporting information. [file JOR-40-1097-s002.docx]

**Supplementary Table 2. Maximum load for anterior, middle, and posterior lateral meniscus (LM).**

|  | **Maximum load (N)** | | |
| --- | --- | --- | --- |
|  | **Anterior** | **Middle** | **Posterior** |
| **Intact** | 0.54  (0.49~0.59) | 0.50  (0.43~0.57) | 0.55  (0.52~0.59) |
| **Meniscectomy** | 0.49  (0.43~0.55) | 0.43  (0.31~0.55) | 0.51  (0.42~0.59) |
| **Extrusion** | 0.083^ab^  (-0.015~0.18) | 0.054^abd^  (-0.025~0.13) | 0.019^ae^  (-0.018~0.056) |
| **Centralization with 1 anchor** | 0.32^c^  (0.21~0.44) | 0.28  (0.13~0.44) | 0.16^c^  (0.055~0.27) |
| **Centralization with 2 anchors** | 0.47  (0.43~0.51) | 0.45  (0.32~0.59) | 0.37  (0.24~0.50) |
| **Centralization with advancement** | 0.52  (0.49~0.55) | 0.55  (0.52~0.58) | 0.43  (0.29~0.56) |

Average values with 95% CI for 6 samples are shown.

^a^ p < 0.05 between the Intact group and the Extrusion group

^b^ p < 0.05 between the Centralization-ad group and the Extrusion group

^c^ p < 0.05 between the Intact group and the Centralization-1 group

^d^ p < 0.05 between the Centralization-2 group and the Extrusion group

^e^ p < 0.05 between the Meniscectomy group and the Extrusion group
