# Supplementary material for: Biomechanical analysis of a centralization procedure for extruded lateral meniscus after meniscectomy in porcine knee joints
Source: J Orthop Res. 2021 Aug 5;40(5):1097–103. doi: 10.1002/jor.25146 (PMC9292650; doi:10.1002/jor.25146)
Supplement: Supplementary file 3 — Supporting information. [file JOR-40-1097-s001.docx]

**Supplementary Table 3. Average contact pressure for anterior, middle, and posterior lateral meniscus (LM).**

|  | **Average contact pressure (Pa)** | | |
| --- | --- | --- | --- |
|  | **Anterior** | **Middle** | **Posterior** |
| **Intact** | 0.13  (0.12~0.15) | 0.090  (0.074~0.11) | 0.092  (0.075~0.11) |
| **Meniscectomy** | 0.11  (0.086~0.13) | 0.10  (0.077~0.13) | 0.11  (0.082~0.13) |
| **Extrusion** | 0.026^ab^  (0.001~0.052) | 0.014^bd^  (-0.001~0.030) | 0.004^bdf^  (-0.004~0.012) |
| **Centralization with 1 anchor** | 0.070^c^  (0.044~0.096) | 0.063^e^  (0.033~0.093) | 0.039  (0.020~0.058) |
| **Centralization with 2 anchors** | 0.11  (0.092~0.13) | 0.11  (0.072~0.16) | 0.078  (0.052~0.11) |
| **Centralization with advancement** | 0.13  (0.11~0.14) | 0.14  (0.10~0.18) | 0.097  (0.067~0.13) |

Average values with 95% CI for 6 samples are shown.

^a^ p < 0.05 between the Intact group and the Extrusion group

^b^ p < 0.05 between the Centralization-ad group and the Extrusion group

^c^ p < 0.05 between the Intact group and the Centralization-1 group

^d^ p < 0.05 between the Centralization-2 group and the Extrusion group

^e^ p < 0.05 between the Centralization-ad group and the Centralization-1 group

^f^ p < 0.05 between the Meniscectomy group and the Extrusion group
