# Supplementary material for: Biomechanical analysis of a centralization procedure for extruded lateral meniscus after meniscectomy in porcine knee joints
Source: J Orthop Res. 2021 Aug 5;40(5):1097–103. doi: 10.1002/jor.25146 (PMC9292650; doi:10.1002/jor.25146)
Supplement: Supplementary file 4 — Supporting information. [file JOR-40-1097-s003.docx]

**Supplementary Table 4. Average contact pressure for the lateral tibial cartilage.**

|  | **Average contact pressure (Pa)** |
| --- | --- |
| **Intact** | 0.085  (0.046~0.12) |
| **Meniscectomy** | 0.14  (0.11~0.17) |
| **Extrusion** | 0.23^ab^  (0.21~0.26) |
| **Centralization with 1 anchor** | 0.21^c^  (0.18~0.24) |
| **Centralization with 2 anchors** | 0.16  (0.11~0.20) |
| **Centralization with advancement** | 0.11  (0.060~0.16)) |

Average values with 95% CI for 6 samples are shown.

^a^ p < 0.05 between the Intact group and the Extrusion group

^b^ p < 0.05 between the Centralization-ad group and the Extrusion group

^c^ p < 0.05 between the Intact group and the Centralization-1 group
